# Myelin oligodendrocyte glycoprotein antibody-associated optic neuritis and myelitis in COVID-19: a case report and a review of the literature

**DOI:** 10.1186/s41983-022-00496-4

**Published:** 2022-05-31

**Authors:** Mark A. Colantonio, Divine C. Nwafor, Shruti Jaiswal, Ashish K. Shrestha, Mahmoud Elkhooly, Steven Rollins, Sijin Wen, Shitiz Sriwastava

**Affiliations:** 1 School of Medicine, West Virginia University, Morgantown, WV USA; 2 Kathmandu Medical College Teaching Hospital, Kathmandu, Nepal; 3 Department of Neuropsychiatry, Minia University, Minia, Egypt; 4 Department of Biostatistics, West Virginia University, Morgantown, WV USA; 5 West Virginia Clinical and Translational Science Institute, Morgantown, WV USA; 6 Department of Neurology, Rockefeller Neuroscience Institute, West Virginia University, One Medical Center Dr., Suite 1310, Morgantown, WV 26506-9180 USA

**Keywords:** COVID-19, SARS-CoV-2, MOG, Optic neuritis, Myelitis, CSF, MRI in COVID-19

## Abstract

**Background:**

Our case explored the spectrum of autoimmune and infectious neurological complications of Coronavirus Disease 2019. In addition, we also reviewed and discussed clinical features, neuroimaging, CSF findings, and outcomes in patients with COVID-19-associated Myelin Oligodendrocyte Glycoprotein Antibody Disorder (MOGAD) CNS inflammatory disorder.

**Case presentation:**

Here we presented a case of post-Coronavirus Disease 2019 infection Myelin Oligodendrocyte Glycoprotein Antibody Disorder in a 41-year-old male who presented with gait instability, urinary retention, and confusion. Workup done in hospital showed transverse myelitis in cervical spine region and left optic neuritis. Laboratory findings showed Myelin Oligodendrocyte Glycoprotein-IgG antibodies were positive in serum (1:100), suggestive of post-COVID Myelin Oligodendrocyte Glycoprotein Antibody Disorder.

**Conclusion:**

To our knowledge, this is the first comprehensive case report and the literature review that includes the clinical features, neuroimaging, CSF findings, and outcomes in COVID-19-associated Myelin Oligodendrocyte Glycoprotein Antibody Disorder.

## Introduction

The novel coronavirus has led to a myriad of complications, including central (CNS) and peripheral (PNS) nervous system dysfunction [1–5]. Coronavirus (SARS-CoV-2) neuropathogenesis is characterized by its hematogenous affinity for Angiotensin-converting enzyme 2 (ACE2) receptors localized to brain endothelial cells and retrograde spread via the glossopharyngeal, vagus, and olfactory nerves [6, 7]. Furthermore, the presence of ACE2 receptors in neurons and glial cells suggests a detrimental role of SARS-CoV-2 on CNS function [7].

Reports of Coronavirus Disease 2019 presenting as Acute Disseminated Encephalomyelitis (ADEM) [8], Myelin Oligodendrocyte Glycoprotein Antibody Disorder (MOGAD) [9], Miller Fisher syndrome [10], and Guillain–Barré syndrome [5] are critical examples of how SARS-CoV-2 may affect proper CNS function due to an aberrant immune response. MOG-IgG's target MOG expressed in oligodendrocytes, which serve as a cellular receptor, adhesion molecule, or regulator of microtubule stability. The pathological effect of MOG-IgG relies on the ability of antibodies to enter the CNS. Thereafter, MOG-IgG-associated neuroinflammation is mediated by T cells and complement-fixing antibodies, ultimately presenting as optic neuritis, transverse myelitis, and (ADEM) [11, 12].

We reported a Myelin Oligodendrocyte Glycoprotein Antibody Disorder case in a 41-year-old male who previously had mild Coronavirus Disease 2019 symptoms based on the ATS/IDSA guidelines [13]. The patient initially presented with transverse myelitis followed by left optic neuritis. Furthermore, we retrospectively discuss the various manifestations, relevant neuroimaging, and cerebrospinal fluid markers of Coronavirus infectious disease-2019 (nCov) associated with MOG-IgG reported to date.

## Case presentation

A 41-year-old male tested positive for the Coronavirus Disease 2019 virus in early November of 2020. Initially, he recovered at home; however, he began to deteriorate, reporting whole-body shivering, confusion, paresthesia, gait instability, and urinary retention 16 days post-COVID diagnosis. His medical comorbidities included hypertension, ethanol, and methamphetamine substance abuse. On examination, the patient appeared diaphoretic, uncomfortable, and was slow to respond to questioning. Initial workup revealed elevated BUN/Creatinine ratio of 24 (range 10:1–20:1), prothrombin time 14.2 secs (range 9.1–13.9), INR 1.23 secs (range 0.80–1.20), venous pH 7.44 (range 7.31–7.41), lactate 1.4 mmol/L (range 0.0–1.3), venous bicarbonate 27 mmol/L (range 22–26), WBC count 18.3 × 10^3^/µL (range 3.7–11.0 × 10^3^), CRP inflammatory maker 9 mg/L (range > 8), neutrophil 14.15 × 10^3^ (range 1.50–7.70 × 10^3^) and PO_2_ 30 mm/Hg (range 35–50), and creatine kinase 23 U/L (range 45–225).

**Fig. 1:**
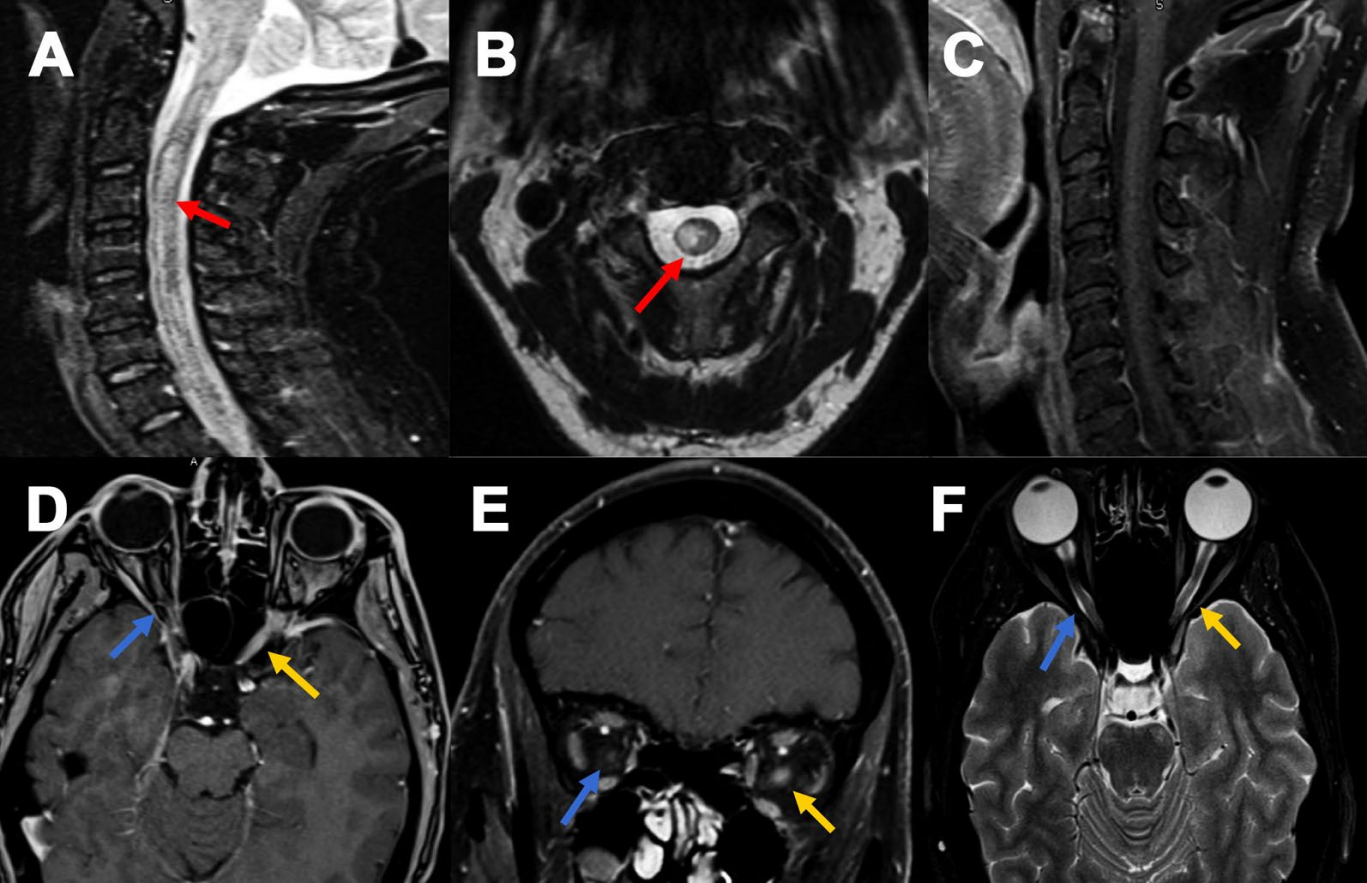
MRI sagittal STIR images (**A**) of the cervical spine reveal ill-defined long segment hyperintensity with prominent cord expansion C2–C4 (red arrow); **B** shows axial cut with cord signal alteration at C3 (red arrow), and **C** post-contrast sagittal image showed no abnormal enhancement. MRI orbit Axial T1-weighted fat suppression post-contrast (**D**) and coronal images (**E**) reveals abnormal enhancement of left optic nerve pre-chiasmatic (intracanicular); (yellow arrow) with corresponding hyperintense on axial T2 weighted images (**F**) (yellow arrow) with no abnormality of the right optic nerve (blue arrow)

Upon admission, the patient underwent magnetic resonance imaging of the spine and brain with and without (w/wo) contrast. MRI of the spine revealed abnormal T2 hyperintensity from C2–C4 without post-contrast enhancement. These results were suggestive of longitudinally extensive transverse myelitis and post-infectious transverse myelitis (Fig. 1). Subsequent lumbar puncture revealed polymorphonuclear leukocytes with an absence of organisms and neutrophilic predominance. CSF revealed 45/µL nucleated cells (range 0–5), 90% lymphocytic predominance, 116 mg/dL protein (range 15–50), 37 mg/dL glucose (range 50–80), 0 oligoclonal bands, and IgG index 0.48. CSF and serum NMO Anti-AQP4 AB were negative, and an MOG test was not performed. For possible meningoencephalitis, the patient was treated with IV ceftriaxone, vancomycin, ampicillin, and acyclovir. On day 6 of admission, a repeat brain MRI suggested a new T2-FLAIR lesion of the left corona radiata and right parietal subcortical white matter. Antibiotics were discontinued as CSF was negative for viral PCR; however, the patient remained confused and lethargic. IV Solumedrol 1 g/day for 5 days was administered, and possible post-COVID CNS inflammatory disorder was suspected.

On day 7 of admission, a follow-up lumbar puncture revealed several polymorphonuclear leukocytes with an absence of organisms and a lymphocytic predominance. CSF analysis yielded 23/µL nucleated cells, protein 62 mg/dL and glucose 44 mg/dL. The patient's mental status improved significantly with the administration of Solumedrol. The diagnosis of post-infectious COVID-19 encephalomyelitis was made, and the patient was discharged 22 days after admission and instructed to follow-up with a neurologist in the next 3 months. At this time, the patient was stable without deficits.

Six months later, the patient returned with complaints of a mild left-sided headache and left-sided retro-orbital pain with visual blurriness occurring over 1 week. The patient was initially seen in the ED, and a workup for possible demyelinating lesions was recommended. Examination of visual acuity in both eyes showed impaired left visual acuity (20/70), right 20/25, and left afferent pupillary defect (APD). Intraocular pressure and extraocular muscles (EOM) were within normal limits and intact. Ophthalmology recommended the patient for orbital MRI with/without contrast for evaluation of potential optic nerve lesions. Brain and orbital MRI w/wo contrast revealed pre-chiasmatic (intracanicular) enhancement of the left optic nerve, suggestive of optic neuritis (Fig. 1). The patient’s concerning encephalopathy, optic neuritis, spinal, and brain lesions raised concern for neurological inflammatory post-COVID vaccination sequala. Hence, CNS inflammatory marker MOG-IgG antibody levels were sent out and tested commercially at the Mayo Clinic via live-cell fluorescent-activated cell sorting assay. MOG-IgG antibodies were positive in serum (1:100), suggestive of post-COVID MOGAD syndrome. Neurology was then consulted for further workup based on the patient’s history of prior CNS lesions and current complaint of left-sided headache and left eye pain. Per neurology recommendations, a repeat MRI of the cervical and thoracic spine w/wo contrast was ordered to rule out multiple sclerosis (MS). MRI imaging was unremarkable and showed no new or enhancing lesion, with an absence of demyelinating lesions in the thoracic spinal cord. Brain MRI revealed decreased conspicuity of previously visualized white matter lesions. Although the patient was admitted, he was started on IV solumedrol (1 g/day for 3 days) and prescribed oral prednisone 1250 mg daily for an additional 2 days to be taken at home following discharge. Although admitted, the patient was scheduled to receive five cycles of PLEX therapy; however, on cycle 3/5, the patient became septic, and PLEX was held in the setting of bacteremia and thrombophlebitis due to a port infection. The remaining PLEX cycles were deferred due to sepsis. The patient stated he had significant improvement of his optic neuritis and visual acuity after receiving 3/5 PLEX cycles and concurrent solumedrol. Following resolution of the patient’s acute bacteremia, he was instructed to return in 1 month for a follow-up visit.

At the patient’s follow-up visit with neurology, the patient reported that his vision had significantly improved, and he is currently stable since his last admission. He no longer has pain and stated his visual acuity had returned near to baseline. Per neurology recommendations and infectious disease clearance, the patient will continue monthly IVIG therapy for suspected post-COVID MOGAD optic neuritis.

## Discussion

Myelin Oligodendrocyte Glycoprotein Antibody Disorder is an inflammatory demyelinating condition of the central nervous system characterized by a monophasic or relapsing neurological dysfunction (optic neuritis, myelitis, and brainstem encephalitis). The findings specific to MOGAD include seropositive MOG-IgG antibodies, and frequently, CNS demyelination via MRI. These findings are MOGAD specific and do not meet diagnostic criteria for MS or other known neuroinflammatory conditions [11, 12, 14].

According to the literature, almost 50% of the patients diagnosed with Myelin Oligodendrocyte Glycoprotein Antibody Disorder present with myalgia, fever, respiratory symptoms, and gastrointestinal symptoms [15, 16]. The post-infectious state of MOGAD may be due to herpes simplex virus, cytomegalovirus, Borrelia, and Epstein–Barr virus infections [16–19]. Several cytokines and inflammatory markers have been implicated to play a role in the post-infectious state and development of MOG-IgG antibodies, including CRP, d-dimer, IL-6, 7, 19, GCSF, IP 10, MCP1.MIP1A and TNF alpha [20]. However, the mechanism contributing to the MOG-IgG antibody is unclear [11].

Interestingly, the recent data suggest a role for Coronavirus Disease 2019 in Myelin Oligodendrocyte Glycoprotein Antibody Disorder relapse [21]. COVID-19-associated MOGAD relapse is likely from the host reaching a threshold, leading to production of MOG-IgG1B-cell [22–24]. More importantly, these findings demonstrate that the SARS-CoV-2 virus may impact disease exacerbation in other relapsing CNS inflammatory disorders [21].

Among initial and relapsing presentations of Myelin Oligodendrocyte Glycoprotein Antibody Disorder, optic neuritis is the most common in adults, ranging between 54 and 60%. A unilateral lesion is seen in 22–40% and bilateral lesion seen in 22–42% [15, 16, 25]. Other clinical manifestations include myelitis, seen in 22–36% of cases, and symptoms of brainstem and encephalopathy syndrome. However, symptomatic presentation of MOGAD appears to be age dependent, as a child will most commonly present with ADEM [15, 26].

Myelin Oligodendrocyte Glycoprotein Antibody Disorder can be further differentiated from other neurological inflammatory conditions based on cerebrospinal fluid findings. MOGAD patients have been shown to yield pleocytosis in 33–66%, and Cobo-Calvo and his colleagues demonstrate it to be more common among children [27]. CSF protein elevation is only found in 27–37% and 11–22% cases can have presence of oligoclonal bands. Differences in CSF composition were further explored in our literature review. Fisher’s exact test was used to assess alterations in CSF composition, including protein, cell count, lymphocytes in post-COVID MOGAD patients. We showed no significant differences in protein, cell count, and lymphocytes were found by age (50+ versus 50−), gender, COVID-19 severity, and fatality, respectively (refer to Table 1).

**Table 1:** CSF findings in patients with post-COVID MOGAD disease

Variables	CSF protein				CSF cell count				CSF lymphocyte %			
	Low (≤ 45)	High (> 45)	*N* (%)	*p*	No (≤ 5)	Yes (> 5)	*N* (%)	*p*	≤ 50%	> 50%	*N* (%)	*p*
Age												
50+	0 (0)	1 (100)	1 (14.3)	0.4	0 (0)	1 (100)	1 (20)	1	No CSF	No CSF		NA
< 50	4 (67)	2 (33)	6 (85.7)		2 (50)	2 (50)	4 (80)		3(75)	1(25)	4(100)	
Gender												
Female	1 (50)	1 (50)	2 (28.6)	1	1 (100)	0 (0)	1 (20)	0.4	1 (100)	0 (0)	1 (25)	1
Male	3 (60)	2 (40)	5 (71.4)		1 (25)	3 (75)	4 (80)		2 (67)	1 (33)	3 (75)	
COVID-19 severity^a^												
Non-severe	3 (50)	3 (50)	6 (85.7)	1	1 (25)	3 (75)	4 (80)	0.4	2 (67)	1 (33)	3 (75)	1
Severe	1 (100)	0 (0)	1 (14.3)		1 (100)	0 (0)	1 (20)		1 (100)	0 (0)	1 (25)	
Fatality												
Non-fatal	4 (57.1)	3 (42.9)	7 (100)	NA	2 (40)	3 (60)	5 (100)	NA	3 (75)	1 (25)	4 (100)	NA
Fatal	0	0	0		0	0	0		0	0	0	

*CSF* cerebrospinal fluid, *NA* not applicable

*P* values were calculated from Fisher’s exact test. All observations were non-fatal and thus a Fisher’s exact test was not available between fatality and the outcomes of interest

^a^Severity based on the IDSA/ATS guidelines

Shared similarities were noted in our case report and published literature. For instance, the patient described in this report initially presented with paresthesia and gait disturbances that improved with Solumedrol. 6 months later, the patient presented with deteriorating symptoms and complaints of optic neuritis. This progression is consistent with MOGAD’s relapsing symptomatic clinical presentation [15, 26]. Furthermore, MRI imaging studies in our patient were consistent with post-infectious transverse myelitis and optic neuritis. Likewise, MOG-IgG antibody titers were elevated (1:100). Taken together, the clinical presentation, imaging studies, and serological analysis seen in our patient strongly supported findings consistent with typical MOGAD presentation [11, 12, 14]. Literature also suggests pleocytosis is a unique and consistent finding in the CSF, as shown in our patient presentation [28].

In addition to our described case report, we conducted a literature review to examine findings from published Myelin Oligodendrocyte Glycoprotein Antibody Disorder data. Table 2 describes MOGAD cases that showed a correlation between COVID-19 and MOGAD. After a comprehensive literature review, 12 cases were identified, with 11 cases fulfilling the diagnostic criteria for MOGAD based on MOG encephalomyelitis: international recommendations on diagnosis and antibody testing [14]. Regarding MOG-IgG Ab, all 11 cases of MOGAD were found to be MOG-IgG Ab serum positive, while only a single case by Khan and his colleagues tested CSF for MOG-IgG Ab and was found to be negative [28] (refer to Table 2). Symptomatic presentation of MOGAD was consistent. Among the 12 reviewed cases, eight presented with symptoms of optic neuritis, as reported by Sawalha and his colleagues, Zhou and his colleagues, Khan and his colleagues, Sardar and his colleagues, Kogure and his colleagues, and de Ruijter and his colleagues, most commonly involving the pre-chiasmal optic nerve [9, 28–31]. Other patient presentations included altered mental status and seizure disorder by Vraka and his colleagues. and Peters and his colleagues, whereas Jumah and his colleagues reported a case of paraplegia [26, 32, 33].

**Table 2:** Study origin, demographics, CSF, MRI findings, severity and outcomes in COVID-19 and MOG-associated disease

Author/country	Patient age/gender	Time duration from COVID-19 to neurological symptom onset	Time duration from COVID-19 to MOG AB positive	Co-morbidity	Neurological presentation	CSF findings	Serum/CSF AQP4, and ANTI-MOG Ab	MRI findings	Diagnosis	Management	Outcomes	Severity based on IDSA/ATS^a^
Sawalha et al./USA	44/M	2 weeks	3 weeks	None	Bilateral eye pain and vision loss	CSF WBC 3/mm^3^, protein 50 mg/dL, glucose 88 mg/dL^b^ OCB negative	Serum AQP4 negative, CSF AQP4 NA, serum MOG Ab positive, CSF MOG Ab NA	Brain MRI showed enhancement in the right more than the left optic nerve suggestive of optic neuritis although no other abnormalities were noted in brain, cervical, or thoracic spine	MOG antibody-associated optic neuritis	IVMP * 5 days Tapering Prednisolone	Not fatal	Not sever
Zhou et al./USA	26/M	< 1 week	1 day	None	Sequential vision loss first affecting the left eye, then the right eye 3 days later	CSF WBC 55/mm^3^, protein 31 mg/dL, glucose-57 mg/dL^b^ OCB mirror	Serum AQP4 negative, CSF AQP4 NA, serum, MOG Ab positive, CSF MOG Ab NA	MRI of the brain and orbits uniform enhancement and thickening of both optic nerves with non-specific focal lesion in left occipital area. MRI spine focal patchy lesion in C and T spine with enhancement	MOG associated optic neuritis and myelitis	IVMP * 5 days Tapering Prednisolone	Not fatal	Not sever
Khan et al./India	11/M	< 1 week	2 weeks	None	Rapidly progressive loss of vision	CSF WBC 55/mm^3^, protein normal, glucose normal^b^ OCB negative	Serum AQP4 negative, CSF AQP4 NA Serum MOG Ab positive, CSF MOG Ab negative	MRI orbit bilateral asymmetrical optic neuritis, with enhancement of the optic nerve sheath in the right orbit MRI brain and spine normal	MOG associated optic neuritis	IVMP * 5 days Tapering Prednisolone	Not fatal	Not sever
Sardar et al./Qatar	38/F	2 weeks	NA	Diabetes Migraine Obesity Obstructive sleep apnea Gastritis	Headache Diminution of vision With painful eye movement	CSF WBC normal, protein normal, glucose-normal^b^ OCB-NA	Serum AQP4 NA, CSF AQP4 NA, Serum MOG Ab NA, CSF MOG Ab NA	MRI orbit T2 signal bilateral optic nerve and enhancement	Seronegative NMOSD and IIH	IVMP for 5 d, IVIG * 5 days PLEX Acetazolamide	Not fatal	Not sever
Zorić et al./Serbia	63/M	4 weeks	11 weeks	Diabetes	Headache Left eye visual loss	CSF NA	Serum AQP4 positive, CSF AQP4 NA, serum MOG Ab positive, CSF MOG Ab NA	MRI brain microangiopathic and a neuroglial cyst on the right temporal side, and normal appearing orbits and optic nerves. MRI C and T spine no abnormal cord signal	MOG associated optic neuritis	IVMP * 5 days Tapering Prednisolone	Not fatal	Not sever
Pinto et al./UK	44/F	1 week	30 days	None	Mild expressive and receptive dysphasia, visual and sensory inattention	CSF WBC 13/mm^3^, protein 507 mg/dL, glucose-52 mg/dL^b^ OCB negative	Serum AQP4 negative, CSF AQP4 NA, serum MOG Ab positive, CSF MOG Ab NA	MRI brain hyperintensity within bilateral periventricular area extending left temporal and occipital horns and into the subcortical deep white matter bilateral. There was perivascular enhancement within the lesions, and MRA was normal. MRI the spine normal. MRI orbit not reported	CNS inflammatory vasculopathy with anti-MOG	IVMP * 5 days Tapering Prednisolone PLEX * 5 Sessions	Not fatal	Not sever
Vraka et al./UK	13m/F	1 week	4 days	None	Altered consciousness, seizures	CSF WBC 10/mm^3^, protein 31 mg/dL, glucose-84 mg/dL^b^ OCB negative	Serum AQP4 negative, CSF AQP4 NA, serum MOG Ab positive, CSF MOG Ab NA	MRI brain hyperintensity in the splenium of the corpus callosum with associated diffusion restriction and high signal in the thalami and pons MRI spine normal	ADEM with anti-MOG	IVMP, acyclovir, levetiracetam	Not fatal	Severe
Kogure et al./Japan	47/M	NA	2 days	Recurrent Paranasal sinusitis Adrenal resection	Left eye pain Visual loss	CSF WBC normal, protein normal, glucose normal^b^ OCB-NA	Serum AQP4 negative, CSF AQP4 NA, serum MOG Ab positive, CSF MOG Ab negative	MRI orbit revealed the bilateral uniform enhancement along with optic nerve sheaths	MOG associated optic neuritis	IVMP * 3 days Tapering Prednisolone	Not fatal	Not sever
Jumah et al./USA	61/M	1 week	1 day	None	Retention sensory Level at T7, paraplegia	CSF WBC 279/mm^3^, protein 106 mg/dL, glucose NA^b^ OCB negative	Serum AQP4 negative, CSF AQP4 NA, serum MOG Ab positive, CSF MOG Ab NA	MRI showed T2 hyperintense lesions of variable length in the mid-thoracic spinal cord, while brain MRI was unremarkable	MOG-antibody myelitis/HHV myelitis	IVMP, PLEX, ganciclovir	Not fatal	Not sever
de Ruijter et al./Netherland	15/M	> 1 week	NA	None	Subacute bilateral visual loss over the course of 7 day	CSF WBC normal, protein normal, glucose normal^b^ OCB-NA	Serum AQP4 negative, CSF AQP4 NA, Serum MOG Ab positive, CSF MOG Ab NA	MRI orbit revealed a bilateral edematous optic nerve lesion	MOG associated optic neuritis	IVMP * 3 days	Not fatal	Not sever
Woodhall et al./UK	39/F	< 1 week	6 days	MOGAD	Painful progressive right visual loss consistent with optic neuritis	CSF NA	Serum AQP4 negative, CSF AQP4 NA, serum MOG Ab positive, CSF MOG Ab NA	MRI progression of right optic nerve atrophy and subtle T2 signal hyperintensity	MOG associated relapse optic neuritis	IVMP * 5 days Mycopheno-late, PLEX * 5 Sessions	Not fatal	Not sever
Peters et al./USA	23/M	2 weeks	2 weeks	Childhood non-febrile seizures	Generalized tonic Clonic seizure, slowing Fever	CSF WBC 57/mm^3^, protein 40 mg/dL, glucose-60 mg/dL^b^ OCB negative	Serum AQP4 negative, CSF AQP4 NA, serum MOG Ab positive, CSF MOG Ab NA	MRI brain diffuse left-hemispheric cortical hyperintensity, most pronounced in the left occipital and posterior temporal lobe with leptomeningeal enhancement Spine MRI not reported	MOG-associated encephalitis	IVMP * 5 days	Not fatal	Not sever

*MOGAD* myelin oligodendrocytes glycoprotein antibody disease, *AQP4* aquaporin antibody, *IVIG* intravenous immunoglobulin, *PLEX* plasmapheresis, *IVMP* intravenous methylprednisolone, *MRI* magnetic resonance imaging, *CSF* cerebrospinal fluid, *OCB* oligoclonal bands

^a^Severity based on the Infectious Diseases Society of America IDSA and American Thoracic Society ATS guidelines

^b^Serum glucose not reported or available

In all cases, Myelin Oligodendrocyte Glycoprotein Antibody Disorder-IgG antibody levels were measured to diagnose Myelin Oligodendrocyte Glycoprotein Antibody Disorder related to Coronavirus Disease 2019 sequela. In 4 cases, MOG antibody tested positive between 2 and 4 weeks [28, 30, 32, 34] whereas Žorić and his colleagues. reported a case where MOG-IgG was positive more than 4 weeks after SARS-CoV-2 RT-PCR positive test [35]. The remaining cases of MOG-IgG positivity were reported within 1 week of the initial COVID-19 positive test (refer to Table 2). Based on this data, MOGAD should not be dismissed solely of the amount of time passed between the initial COVID-19 positive test and subsequent MOG-IgG testing.

Cerebrospinal fluid was also analyzed to distinguish Myelin Oligodendrocyte Glycoprotein Antibody Disorder from other neuroinflammatory diseases further. More than half of the CSF in the studied cases (6 out of 10) revealed pleocytosis. In contrast, only 3 cases by Sawalha and his colleagues, Pinto and his colleagues, and Jumah and his colleagues. reported high protein levels in the CSF [30, 33, 34]. Oligoclonal bands in CSF were assessed in 8 cases; however, no cases had unique isolated bands in CSF (refer to Table 2).

Upon further analysis of the cases, two-third of cases (7/12) showed T2 hyperintensity and post-contrast enhancement in the prechiasmal optic nerves with sparing of chiasma and optic tract. Woodhall and his colleagues. was the only case to report unilateral optic nerve lesions, with all others showing bilateral optic nerve involvement [21]. In addition, Zhou and his colleagues reported a case of MOGAD with findings of optic neuritis and myelitis. Subsequent MRI showed non-enhancing, non-specific periventricular T2 hyperintensity adjacent to the occipital horn of the right lateral ventricle with patchy T2 hyperintensities in the lower cervical and upper thoracic spinal cord. Mild central thickening and gadolinium enhancement were also observed [9]. However, Jumah and his colleagues. reported a case of MOGAD with subsequent spinal MRI imaging revealing the spine revealing non-enhancing T2 hyperintense lesions of variable length in the mid-thoracic spinal cord [33]. Peters and his colleagues. reported encephalitis with MRI finding of T2 hyperintensity in left occipital and posterior temporal lobe with leptomeningeal enhancement [32]. Moreover, Vraka and his colleagues reported MRI findings of bilateral widespread white matter high-signal abnormalities, including the splenium of the corpus callosum with associated diffusion restriction and high signal in the thalami and pons in a diagnosed MOGAD 13-month-old female [26] (refer Table 2).

The studied cases used similar treatment for Myelin Oligodendrocyte Glycoprotein Antibody Disorder. All cases recovered after initiation of intravenous methylprednisolone. Only 4 cases by Sardar and his colleagues, Pinto and his colleagues, Jumah and his colleagues, and Woodhall and his colleagues were treated with plasma therapy due to the slow and partial improvement in symptoms [21, 29, 33, 34]. The above therapies proved to yield a favorable outcome with the treatment of MOG antibody disorder.

One limitation of this review study was the small sample size used. A future study should be considered with a larger sample size when there is a greater prevalence of concurrent COVID-19 related MOGAD. This will help further explore similarities and differences among patient symptom presentations, imaging similarities, CSF analysis, and treatment outcomes.

## Conclusion

Our case explored the spectrum of autoimmune and infectious neurological complications of Coronavirus Disease 2019. In addition, we also reviewed and discussed clinical features, neuroimaging, CSF findings, and outcomes in patients with COVID-19-associated MOGAD CNS inflammatory disorder. Our cases provided insight regarding the need to test specific demyelinating antibodies, such as MOG-IgG in the setting of a suspicious clinical picture, such as longitudinally extensive myelitis or severe optic neuritis, especially in the setting of a concurrent or previous COVID-19 infection. Future research should focus on early diagnosis of MOGAD and testing modalities, such as MRI imaging, serum, and CSF analyses. In light of this, we believe this review will be an essential aid to future studies on CNS inflammatory disorders, such as MOGAD in relation to COVID-19 and provide helpful information for researchers and registries to collect future data on MOGAD and COVID-19.

## Data Availability

Data was extracted from the articles published in PUBMED, Google Scholar, Scopus. This will be provided on request.

## Abbreviations

COVID-19: Coronavirus infectious disease-2019
nCov: Novel Coronavirus
SARS-CoV-2: Severe Acute Respiratory Distress Syndrome coronavirus 2
CSF: Cerebrospinal fluid
CNS: Central nervous system
PNS: Peripheral nervous system
ACE2: Angiotensin-converting enzyme 2
RT-PCR: Reverse transcription polymerase chain reaction
IDSA/ATS: Infectious Diseases Society of America/American Thoracic Society
AHNE: Acute hemorrhagic necrotizing encephalitis
AHLE: Acute hemorrhagic leukoencephalitis
ADEM: Acute Disseminated Encephalomyelitis
MRI: Magnetic resonance imaging
CLOCC: Cytotoxic lesion of the Corpus Callosum
MOG: Myelin Oligodendrocyte Glycoprotein
MERS: Mild encephalopathy reversible splenium lesion
MOGAD: Myelin Oligodendrocyte Glycoprotein Antibody Disorder
GBS: Guillain–Barre syndrome
WHO: World Health Organization
IVIG: Intravenous immunoglobulin
IVMP: Intravenous methylprednisolone
PLEX: Plasma exchange/plasmapheresis
